# Retropharyngeal abscess and stridor in infants

**DOI:** 10.1016/S1808-8694(15)30799-0

**Published:** 2015-10-19

**Authors:** Regina Helena Garcia Martins, Emanuel Celice Castilho, Silke Tereza Weber, Graziela de Oliveira Semenzati, Lígia M.P. de Campos

**Affiliations:** 1 Assistant professor, doctorate in surgery, Faculdade de Medicina de Botucatu - UNESP. Head of the phoniatrics and voice outpatient unit. Faculty member of the Otorhinolaryngology Discipline, Universidade Estadual Paulista-Unesp, Botucatu campus; 2 Adjunct professor, physician of the Otorhinolaryngology Discipline, Faculdade de Medicina de Botucatu - UNESP; 3 Adjunct professor, faculty member of the Otorhinolaryngology Discipline, Faculdade de Medicina de Botucatu-Unesp; 4 Medical residence, Otorhinolaryngology Discipline, Faculdade de Medicina de Botucatu - UNESP; 5 Medical residence, Otorhinolaryngology Discipline, Faculdade de Medicina de Botucatu - UNESP

**Keywords:** retropharyngeal abscess, dyspnea, stridor, infants

## INTRODUCTION

Retropharyngeal abscesses generally result from upper aerodigestive infection or trauma, such as accidental swallowing of a foreign body or traumatic orotracheal intubation. Such abscesses occur mostly in children up to age 5 years. Initial symptoms are non-specific, such as irritability, loss of appetite and fever; as the condition progresses, there may be dysphagia, odynophagia, sialorrhea, a “hot potato” (garbled) voice or a muffled cry.^1^, ^2^ The severity of symptoms is directly related with the volume of the abscess; there may be severe respiratory distress.^2^

The presentation may be dramatic, because as the abscess forms in the retropharyngeal space it grows towards the soft palate, base of tongue, posterior pharyngeal wall and larynx, which narrows considerably the airway lumen. This may lead to respiratory failure, especially in nursing babies.

Upon confirmation of the diagnosis of a retropharyngeal abscess, the child should immediately be admitted to hospital for continuous monitoring of vital signs, therapy with endovenous antibiotics, and surgical drainage. Spontaneous rupture of the abscess may lead to massive aspiration of pus and the development of pulmonary empyema, mediastinitis and septicemia. Orotracheal intubation or a tracheotomy is required to assure airway patency.^3^, ^4^, ^5^, ^6^

## CASE REPORT

**Figure 1:**
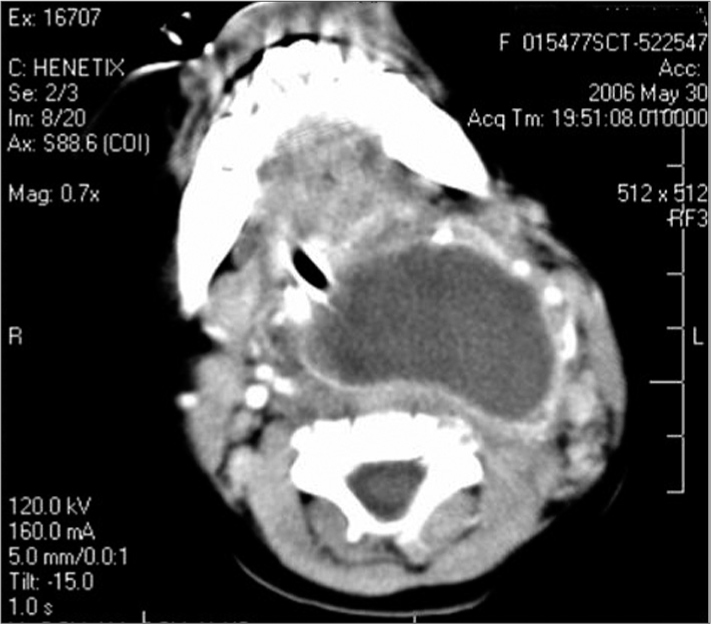
Large retropharyngeal abscess seen in computed tomography (CT).

RVNS, a female child aged 10 months, was admitted to the Emergency Unit; the mother informed that the child had been feverish for the past 20 days, and that there was a yellowish secretion from the nose and nose block. She had sought a clinic in her own town, and the child had been prescribed amoxicillin to be taken during 10 days. Two days after this treatment ended, the child started to breathe and swallow with difficulty; there was also neck enlargement. In the physical examination, the child was fatigued, febrile, tachycardic and tachypneic; there was a stridor and retraction of the furcula, and the child was either awake or drowsy. There was a yellowish secretion in the nasal fossae, the right contour of the neck and the posterior oropharyngeal wall were altered. Oral intubation was done promptly, blood samples were taken for a complete blood count and culture, and endovenous antibiotics (amoxicillin + clavulanate and metronidazole) were started. Computed tomography of the neck revealed a large retropharyngeal abscess (Fig. 1). Surgical drainage was achieved by a transoral approach; there was abundant pus. Cultures of the pus and blood were negative. The child progressed favorably and was extubated on the day after surgery.

## DISCUSSION

The main cause of stridor in children below one year of age is laryngomalacia. Retropharyngeal abscesses - usually secondary to upper airway infections^1^^,^^4^ - are another important cause, given the severity of this condition. The diagnosis is based on the clinical history and the physical examination; it should be supplemented by image exams, such as ultrasound, CT and MRI, which provide information about the extent of the disease and complications. The case above stands out because of the severity of respiratory distress due to the size of the abscess. Additionally, symptoms arose early if we consider that the child was aged less than one year, a period in which a precise diagnosis is usually not made initially, as in this case. Cultures of the oropharyngeal secretion and of blood were negative, since the child was on antibiotics. The usual microorganisms in retropharyngeal abscesses are Streptococcus spp., Staphylococcus spp. and anaerobes.^5^^,^^6^

## FINAL COMMENTS

The retropharyngeal abscess is an important cause of stridor in nursing babies. This is a severe disease that may cause major respiratory distress. Delays in the diagnosis may result in high morbidity and mortality; prompt treatment with medication and surgery is mandatory.
